# Multifaceted Study of *Medicago marina* Essential Oil: Chemical Composition, Antidiabetic, and Phytotoxic Insights via In Vitro and In Silico Approaches

**DOI:** 10.1002/cbdv.202501574

**Published:** 2025-07-31

**Authors:** Marwa Melliti, Assia Hamdi, Mabrouk Horchani, Eliza de Jesus Barros dos Santos, Hichem Ben Jannet, Mohammad Faisal, Abdulrahman A. Alatar, Marcello Iriti, Mozaniel Santana de Oliveirae, Hayet Edziri

**Affiliations:** ^1^ Laboratory of Transmissible Diseases and Biologically Active Substances (LR99ES27) Faculty of Pharmacy University of Monastir Monastir Tunisia; ^2^ Laboratory of Chemical Pharmaceutical and Pharmacological Development of Drugs Faculty of Pharmacy University of Monastir Monastir Tunisia; ^3^ Laboratory of Heterocyclic Chemistry Natural Products and Reactivity (LR11Es39) Medicinal Chemistry and Natural Products Faculty of Sciences University of Monastir Monastir Tunisia; ^4^ Department of Botany & Microbiology College of Science King Saud University Riyadh Saudi Arabia; ^5^ Department of Biomedical Surgical and Dental Sciences University of Milan Milan Italy; ^6^ National Interuniversity Consortium of Materials Science and Technology (INSTM) Firenze Italy; ^7^ Laboratory of Pharmacology of Inflammation and Behavior Programa De Pós‐Graduação em Ciências Farmacêuticas, Institute of Health Sciences Federal University of Pará Belém Brazil

**Keywords:** α‐amylase, allelopathic activity, essential oil, *Medicago marina*, molecular docking

## Abstract

The essential oil of *Medicago marina* was subjected to analysis at vegetative and reproductive stages of development, with a particular emphasis on its chemical composition, α‐amylase inhibition, and allelopathic activities. The results of the gas chromatography and molecular docking studies demonstrated alterations in oil yield and composition throughout the developmental stages. Before blooming, hydrocarbons were the predominant compounds, while monoterpenes were abundant during the reproductive stage, with higher concentrations of eugenol (8.86%) and β‐ionone (17.67%) compared to earlier stages. The α‐amylase activity exhibited variation across the growth stages, with the highest enzyme inhibition (87.33 ± 0.001%) observed at 1.25 mg/mL during the reproductive stage. The essential oil demonstrated pronounced allelopathic effects, markedly reducing radish stem elongation (94.09 ± 0.62%) and root growth (95.71 ± 0.2%). Furthermore, molecular docking analysis confirmed that five principal oil components exhibited strong inhibitory activity against human pancreatic α‐amylase and 4‐hydroxyphenylpyruvate dioxygenase (HPPD). These findings underscore the potential of *M. marina* essential oil as a hypoglycemic and allelopathic agent, influencing seed germination and the growth of radish and lettuce.

## Graphical abstract

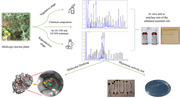

## 1 Introduction

*Medicago marina* L., a plant indigenous to the Mediterranean, is widely distributed in Spain, northern Africa, and parts of central Asia. It flourishes in coastal dunes and islands along the Italian coast, playing a pivotal role in soil stabilization [1]. Notable for its golden yellow flowers, *M. marina* has also garnered attention in traditional Spanish medicine [1]. Plants from the genus *Medicago* have shown promising antidiabetic properties due to the presence of valuable natural compounds [2]. In fact, *Medicago sativa* have been previously proven in traditional medicine as an antidiabetic agent [3].

Essential oils (EOs) are volatile, aromatic liquids produced by different parts of plants [4]. These oils have a long history of application in traditional medicine, particularly for preventing and treating bacterial infections [5, 6, 7]. The composition of EOs is highly variable and influenced by genetic factors, plant parts, and developmental stages [8, 9, 10]. The EO composition of *M. marina* has been reported [1], and previous research has demonstrated that growth stages and harvesting periods significantly affect both the yield and chemical composition of EOs [11].

Another significant global health concern is Type 2 diabetes mellitus (T2DM), a noncommunicable disease characterized by pancreatic β‐cell damage and insulin resistance, resulting in postprandial hyperglycemia [12]. The prevalence of T2DM has been exacerbated by urbanization and increased carbohydrate consumption, resulting in a significant health and economic burden. Pancreatic α‐amylase plays a pivotal role in starch hydrolysis, a key contributor to postprandial hyperglycemia. Therefore, inhibiting enzymes such as α‐amylase and α‐glucosidase represents a potential strategy for controlling diabetes by slowing starch digestion [13]. Nevertheless, the current treatments for T2DM remain inadequate, underscoring the necessity for preventive measures and alternative therapies [14].

To address these challenges, researchers have turned to plant‐based alternatives for the development of new antidiabetic agents. Plant‐derived drugs are frequently more cost‐effective and demonstrate fewer adverse effects than their synthetic counterparts [15]. A previous study reported that *M. rigidula* methanol extract exerted 0.73 ± 0.04 mmol ACAE/g of extract [16].

Plant interactions, both within and across species, play an important role in the structure of plant communities. Allelopathy is a well‐known example of such interactions, the chemical influence of one plant on another, which can result in a good or bad impact either directly or indirectly [17, 18]. Natural plant‐derived substances offer a number of advantages over their synthetic counterparts, including rapid biodegradability, low toxicity to nontarget organisms, and a minimal risk of resistance development [19].

A considerable number of allelochemicals, which are secondary metabolites in plants, are employed as substitutes for synthetic herbicides. Such compounds include alkaloids, tannins, and glycosides [20]. By mimicking or inhibiting plant germination and growth, allelochemicals are considered an eco‐friendly and effective substitute for synthetic herbicides due to their lack of harmful or persistent effects [21]. As a result, investigating the EO of *M. marina* provides for a two‐pronged approach: assessing its α‐amylase inhibitory capability for antidiabetic uses and its allelopathic activity as a natural herbicide. Both effects stem from the plant's chemical defense strategy, implying a common bioactivity mechanism that targets critical enzyme pathways throughout biological domains.

This work represents the first report on the dual biological activities: α‐amylase inhibition and allelopathic effects of *M. marina* EO. The goal of our present study was to examine the biological potential of *M. marina*, a plant species that has not been extensively studied with regard to its medicinal properties. This paper investigates the chemical composition of *M. marina* EO at vegetative and reproductive stages of development and evaluates its allelopathic and α‐amylase properties. In addition, the molecular docking study of the major phytocompounds toward “human pancreatic α‐amylase” “PDB ID: 3BAJ” and 4‐hydroxyphenylpyruvate dioxygenase “HPPD” for allelopathic potential was conducted.

## 2 Results and Discussion

### 2.1 Chemical Composition

The results of this study showed that the oils obtained from the different growth stages had interesting differences in both the chemical composition and the extraction yield. A notable variation was revealed in the qualitative and quantitative composition.

The chemical analysis of *M. marina* revealed significant differences in chemical classes between the vegetative and reproductive stages, indicating alterations in secondary metabolism throughout the plant's developmental stages (Table 1). Monoterpene ketones, for instance, demonstrated a notable increase from 3.468% in the vegetative stage to 23.224% in the reproductive stage, potentially attributable to the heightened biosynthesis of compounds such as (*E*)‐β‐ionone (17.677% in the reproductive stage compared to 1.798% in the vegetative stage). This change suggests an increased need for volatile compounds related to pollinator attraction during the reproductive stage. In addition, phenols demonstrated an increase, rising from 2.614% in the vegetative stage to 21.377% in the reproductive stage. Of particular note were eugenol (8.868%) and methyl eugenol (10.759%), which exhibited prominence in the reproductive stage. These compounds are renowned for their antimicrobial properties and potential role in safeguarding the plant against pathogens. Conversely, aldehydes, which constituted the most abundant chemical class in the vegetative stage (25.445%), exhibited a notable decline to 13.702% in the reproductive stage. Among these, nonanal stood out with a significant reduction from 7.388% in the vegetative stage to 1.513% in the reproductive stage.

**TABLE 1:** The chemical composition of *Medicago marina* essential oil in the vegetative and reproductive stages, relative abundance (%).

Number	RI_L_	Compound	Vegetative	Reproductive
1	833	Furfural	4.313	0.071
2	855	(*E*)‐2‐Hexenal	1.423	0.086
3	894	2‐Heptanone	0.338	0.037
4	903	*n*‐Nonane	0.352	0.567
5	904	5‐Heptanal	2.416	1.019
6	906	(Z)‐2‐Heptenal	0.103	0.899
7	965	Benzaldehyde	0.885	1.695
8	974	Heptanol	0.677	0.212
9	980	(*E*)‐2‐Heptenal	0.125	0.107
10	983	10‐1‐Octen‐3‐one	0.269	0.261
11	985	1‐Octen‐3‐ol	4.978	0.561
12	988	6‐Methyl‐5‐hepten‐2‐one	0.075	0.125
13	996	2‐Pentylfuran	0.072	0.165
14	1005	Octanal	0.714	0.032
15	1017	(*E*,*E*)‐2,4‐Heptadienal	0.391	0.094
16	1038	1,8‐Cineole	0.433	0.276
17	1046	Phenylacetaldehyde	1.155	0.339
18	1066	(*E*)‐2‐Octenal	0.421	0.283
19	1075	1‐Undecene	0.721	0.464
20	1092	Linalool	0.226	0.474
21	1104	Nonanal	7.388	1.513
22	1115	(*E*,*E*)‐2,4‐Octadienal	0.153	0.133
23	1120	Isophorone	0.378	0.345
24	1150	Veratrole	0.136	0.149
25	1158	(*E*,*Z*)‐2,6‐Nonadienal	0.053	0.36
26	1167	(*E*)‐2‐Nonenal	0.019	0.118
27	1175	1‐Nonanol	2.171	0.223
28	1180	2,4‐Dimethylbenzaldehyde	0.213	0.65
29	1190	α‐Terpineol	0.177	0.074
30	1195	1‐Dodecene	0.045	0.159
31	1197	Methyl salicylate	0.275	0.161
32	1203	Safranal	1.721	0.096
33	1208	Decanal	0.588	2.351
34	1226	β‐Cyclocitral	0.193	0.301
35	1255	*p*‐Menth‐4‐en‐3‐one	1.505	0.298
36	1265	(*E*)‐2‐Decenal	0.153	0.668
37	1295	Thymol	0.268	1.072
38	1308	Undecanal	0.586	1.505
39	1310	4‐Vinyl guaiacol	0.096	0.368
40	1316	(*E*,*E*)‐2,4‐Decadienal	1.233	0.869
41	1356	Eugenol	0.25	8.868
42	1385	(*E*)‐β‐Damascenone	0.156	4.337
43	1403	Methyl eugenol	1.589	10.759
44	1406	Longifolene	2.569	1.88
45	1410	(*E*)‐β‐Damascone	0.444	0.865
46	1420	β‐Cedrene	0.896	0.617
47	1455	(*E*)‐Geranylacetone	1.07	1.21
48	1458	α‐Humulene	2.258	4.323
49	1485	(*E*)‐β‐Ionone	1.798	17.677
50	1498	Benzyl tiglate	3.013	0.863
51	1500	*n*‐Pentadecane	3.469	2.605
52	1525	δ‐Cadinene	1.197	1.833
53	1581	Caryophyllene oxide	0.377	0.368
54	1607	5‐Eudesmen‐1‐ol	0.678	0.495
55	1700	*n*‐Heptadecane	0.86	0.43
56	1717	Pentadecanal	1.197	0.514
57	1763	Benzyl benzoate	0.484	0.558
58	1845	Hexahydrofarnesylacetone	1.464	0.228
59	1900	Nonadecane	3.328	0.278
60	1927	Methyl hexadecanoate	1.89	1.408
61	2100	*n*‐Heneicosane	1.644	0.526
62	2120	Phytol	5.638	1.701
63	2200	*n*‐Docosane	0.9	0.437
64	2300	*n*‐Tricosane	1.029	0.463
65	2400	*n*‐Tetracosane	1.609	0.828
66	2500	*n*‐Pentacosane	4.363	0.121
67	2600	*n*‐Hexacosane	2.042	0.692
68	2700	*n*‐Heptacosane	0.455	0.656
69	2800	*n*‐Octacosane	2.646	1.263
70	2900	*n*‐Nonacosane	0.282	0.749
71	3000	*n*‐Triacontane	0.767	0.877
Extraction yield (%)			0.071	0,16
Ketone monoterpenes			3.47	23.22
Phenols			2.61	21.38
Aldehydes			25.45	13.7
Hydrocarbons			23.39	9.93
Sesquiterpenes			7.98	9.52
Esters			5.39	2.83
Ketones			4.38	1.86
Hydrogenated diterpenes			5.64	1.7
Alcohols			7.83	1
Oxygenated monoterpenes			0.84	0.83
Other			0.84	0.79

Hydrocarbons, although also more prevalent in the vegetative stage (23.394%), exhibited a notable decline in the reproductive stage (9.925%), suggesting a potential shift in the focus towards compounds with a more pronounced role in chemical ecology during the reproductive stage. Similarly, alcohols, including 1‐octen‐3‐ol, were more prevalent in the vegetative stage (7.826%) than in the reproductive stage (0.996%). The analysis of *M. marina* EOs identified a total of 71 components, representing 94.4% of the volatile oil composition during the reproductive stage [22]. Our findings indicate notable alterations in the concentrations of major components between the vegetative and reproductive stages, underscoring the dynamic nature of secondary metabolite production during plant development.

For example, the concentrations of specific compounds, including β‐ionone (1.79%), eugenol (0.25%), methyl eugenol (1.58%), and damascenone (0.156%), were significantly higher in the reproductive stage compared to the vegetative stage. The eugenol exhibited a 25‐fold increase, β‐ionone a ninefold increase, and methyl eugenol a sixfold increase. This trend highlights the potential involvement of these metabolites in reproductive processes, including pollinator attraction or protection [23]. It is noteworthy that the relative abundance of monoterpenes increased markedly from 3.46% in the vegetative stage to 23.22% during the reproductive stage, a finding that is corroborated by previous research [24].

These observations are consistent with the hypothesis that ontogenesis affects the biosynthesis and accumulation of active compounds in plants [25]. Such differences are likely attributable to alterations in metabolic pathways as the plant progresses through its developmental stages. For example, our study found that furfural, β‐cedrene, and δ‐cadinene were absent in the vegetative stage, which is consistent with earlier findings [1]. However, phytol, an oxygenated diterpene absent in Italian populations of *M. marina* [1], was found to be abundant in our samples, comprising 5.63% in the vegetative stage and 1.7% in the reproductive stage. The presence of this compound is indicative of both environmental and genetic influences, as phytol has also been reported in other species during vegetative growth stages [26].

The yield of EO was found to be significantly higher during the reproductive phase, with a twofold increase compared to the vegetative stage. This discrepancy may be attributed to a number of factors, including genetic variability, phenological stage, and postharvest handling techniques such as drying and storage [24]. These findings underscore the intricate interrelationship between plant maturity, environmental conditions, and biochemical processes, which collectively influence the EO profile and yield. These results support the hypothesis that secondary metabolite synthesis in *M. marina* is significantly affected by developmental stages and external factors, including habitat and climate [27]. Further molecular studies are required to elucidate the regulatory mechanisms underlying these variations and their ecological significance.

### 2.2 α‐Amylase Inhibition

The findings demonstrate the pronounced inhibitory impact of *M. marina* EO at a concentration of 1.25 mg/mL on α‐amylase activity, as evidenced in Table 2, with IC_50_ values of 0.0126 and 0.0138 mg/mL for the vegetative and reproductive stages, respectively. Compared with the positive control, acarbose, which has an IC_50_ value of 0.015 mg/mL. Furthermore, the inhibitory effect of *M. marina* EO was more pronounced during the reproductive stage (87.33%) compared to the vegetative stage (80.26%), and the percentage of inhibition was slightly higher than that of acarbose (84.93%). The amylase inhibition percentages varied significantly across the growth stages (*p* < 0.05) as confirmed by one‐way ANOVA followed by Tukey's test.

**TABLE 2:** α‐Amylase inhibition activities in *Medicago marina* essential oil sample compared with acarbose as positive control using DNS assay, results are expressed as mean ± SD (*n* = 3).

	*M. marina* essential oils		Acarbose
	Vegetative stage	Reproductive stage	
Percentage of inhibition	80.26^a^ ± 0.0003	87.33^b^ ± 0.001	84.93^b^ ± 0.00
IC_50_ (mg/mL)	0.0126^a^ ± 0.001	0.0138^a^ ± 0.00	0.015^a^ ± 0.000023

*Note*: Means followed by the same letter do not differ significantly depending on Tukey's test (*p* < 0.05).

The bioactivity of *M. marina* EO can be attributed to its rich composition of bioactive compounds during the reproductive stage, including eugenol and β‐ionone [22]. Eugenol, a principal constituent, is renowned for its diverse pharmacological attributes, encompassing local anesthetic, anti‐inflammatory, analgesic, antioxidant, and antibacterial properties [28, 29].

A recent study suggested that eugenol, among major compounds found in *Cinnamomum tamala* EOs, exhibits a dose‐dependent amylase inhibition effect with IC_50_ = 62.53 µg/mL and an in silico assay demonstrated that eugenol binds near the enzyme's active site with a mechanism involving hydrogen bonding and hydrophobic link [30].

Previous in silico findings demonstrated that eugenol had high energy binding to albumin compared to glucose (−Δ2.75 vs. −Δ1.27 kcal/mol) [31]. Another study suggested that α‐glucosidase, α‐amylase, PTP1B, glucokinase, and HK‐II are among the enzymes along the glucose metabolic pathways that may be impacted by components in cinnamon EO, including eugenol, according to in silico molecular docking simulations [32].

Moreover, β‐ionone, another pivotal constituent of *M. marina* EO, has been previously linked to α‐amylase inhibition, as substantiated by investigations on *L. setulosus* EO [33]. This lends further support to the hypothesis that the anti‐α‐amylase activity of *M. marina* oil is a result of a synergistic effect between its components. For comparison, the α‐amylase (95.4%) and α‐glucosidase (98.9%) inhibitory activities of thyme EO were linked to its thymol content [34], underscoring the importance of specific phytochemicals in enzyme inhibition.

The study highlights the potential of *M. marina* EO as an alternative to acarbose, an α‐amylase and α‐glucosidase inhibitor utilized for the management of glucose levels in patients with Type 2 diabetes. Although acarbose is an effective treatment, it frequently results in adverse effects, including flatulence and diarrhea [35]. The modestly decreased IC_50_ values of *M. marina* EO may indicate enhanced efficacy and a reduced incidence of adverse effects, thereby supporting the traditional use of *Medicago* species and paving the way for its application as an antidiabetic agent [3]. Depending on our findings, although the difference may look minor, its therapeutic significance is determined by whether it is adequate to impact postprandial blood sugar levels or enhance clinical outcomes in illnesses such as Type 2 diabetes, and this could guide harvesting decisions or formulation strategies where maximizing α‐amylase is preferred.

In accordance with prior research indicating the hypoglycemic effects of the *M. sativa* species [36], these findings substantiate the therapeutic potential of *Medicago* bioproducts. This study further supports the development of natural, plant‐based treatments for diabetes, emphasizing their efficacy and safety in comparison to synthetic alternatives.

However, the inhibition effect noticed for the EO during the vegetative stage could be explained by the chemical composition as well, in fact, phytol was among the predominant compounds and it is previously demonstrated that phytol found as a major compound in the hexane extract of *Leptadenia lanceolata* has a strong α‐amylase inhibition activity (83%) [37]. In addition, a recent review demonstrated that phytol has been reported to inhibit postprandial blood sugar [38].

Another study indicated that 63% of α‐amylase inhibition was detected for *Calotropis gigantea* EO, where phytol (24%) was the predominant compound [39].

### 2.3 Evaluation of Allelopathic Potential

#### 2.3.1 Effect of *M. marina* EO on Seed Germination

**FIGURE 1:**
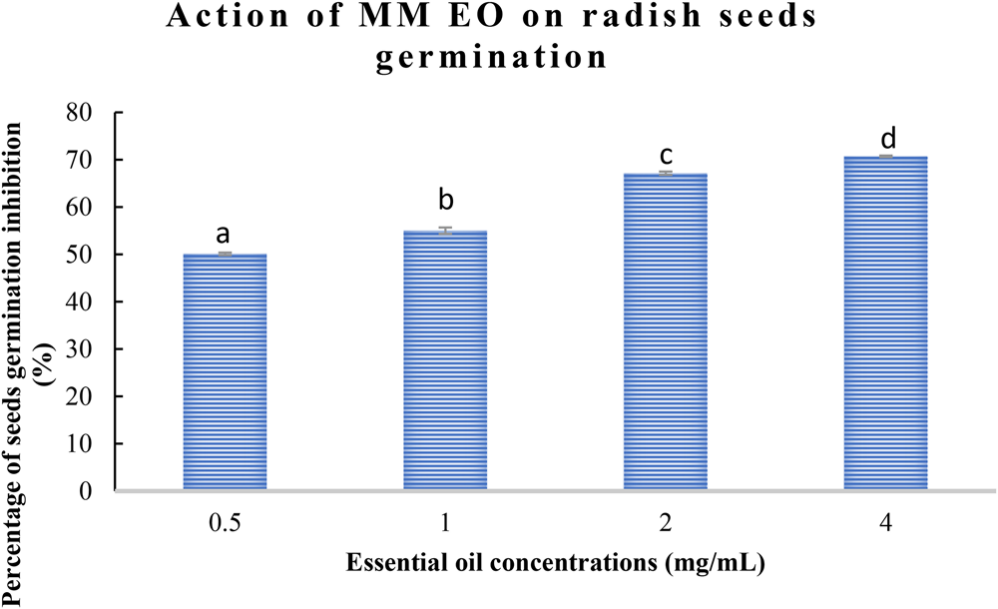
Germination percentage of radish seeds in the presence of *Medicago marina* essential oil at different concentrations. Results are expressed as mean ± SD (*n* = 3) and values shared the same letter indicate no significant difference (*p* < 0.05).

**FIGURE 2:**
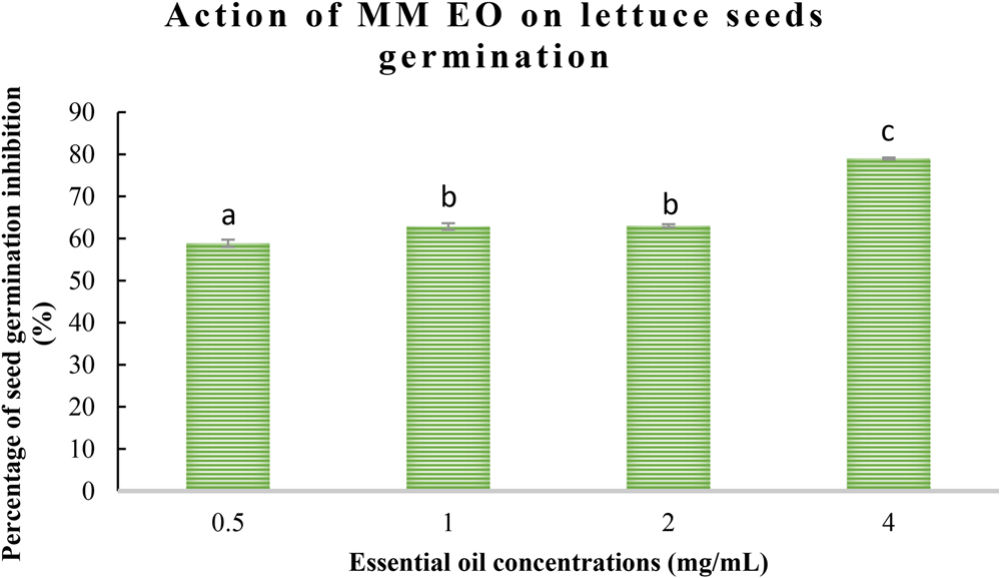
Germination percentage of lettuce seeds in the presence of *Medicago marina* essential oil at different concentrations. Results are expressed as mean ± SD (*n* = 3) and values shared the same letter indicate no significant difference (*p* < 0.05).

The allelopathic activity of *M. marina* EO during the reproductive growth stage was evaluated, with the results presented in Figures 1 and 2. The data demonstrate that *M. marina* EO exerts a significant inhibitory action on the germination and growth of lettuce and radish seeds, with effects becoming more pronounced at higher concentrations.

As illustrated in Figures 1 and 2, the germination rates of both lettuce and radish seeds exhibited a decline as the concentration of *M. marina* EO increased. At the highest concentration (4 mg/mL), a significant decline in germination percentage was observed, reaching 70.71 ± 0.5% for radish seeds and 79 ± 0.9% for lettuce seeds. In contrast, at a low concentration (0.5 mg/mL), an inhibition of 50.1 ± 0.02% and 58.87 ± 0.5% for radish and lettuce seeds was observed, respectively.

One of the most common metrics used to assess allelopathic activity is the inhibition of germination. According to the findings, *M. marina* oil significantly affected germination. For radish, the percentages of germination inhibition rise as EO concentrations rise (70.71 ± 0.2% at a concentration of 4 mg/mL), and the phytotoxicity on the seeds was dosage dependent. The highest inhibition of germination percentage for lettuce seeds was 79 ± 0.2% at a concentration of 4 mg/mL.

To the best of our knowledge, no studies have been reported dealing with the allelopathic activities of *M. marina* EO.

Interestingly, our findings differ from a prior research which has demonstrated that the 80% methanol extract and organic phase (acid hydrolysis) at 10 mg/mL of *M. sativa* significantly enhanced seed germination, with an increase of 92.50 ± 4.00%, compared to the control seed [40].

#### 2.3.2 Effect of *M. marina* EO on Root and Stem Elongation

The results presented in Table 3 indicate a general tendency for an increase in the measured values as the concentration of the substance in question increases. This suggests a concentration–effect relationship. At a concentration of 0.5 mg/mL, the values are lower, with the radish stem treatment exhibiting the highest value (26.29 ± 0.7). As the concentration of the substance increases, the values for the radish and lettuce stems and roots also increase significantly, with the highest value observed in the lettuce roots at the concentration of 4 mg/mL (89.32 ± 1.13).

**TABLE 3:** Inhibitory effects of different concentrations of *Medicago marina* essential oil on roots and stems elongation of radish and lettuce (%).

Concentrations (mg/mL)	Inhibition in relation to control (%)			
	Radish stem	Lettuce stem	Radish root	Lettuce root
0.5	26.29 ± 0.7^b^	16.75 ± 0.1^a^	24.75 ± 0.6^b^	18.87 ± 0.2^a^
1	77.83 ± 0.3^c^	42.60 ± 1.4^c^	25.415 ± 0.3^b^	73.35 ± 9.8^c^
2	87.96 ± 7.5^cd^	78.73 ± 0.74^d^	64.49 ± 0.8^c^	85.67 ± 7.8^d^
4	94.09 ± 0.62^d^	93.64 ± 2^e^	95.71 ± 0.2^c^	89.32 ± 1.13^d^

*Note*: No statistical significance between the samples is presented by the same letter.

In general, radish stems demonstrate the highest values at all concentrations, followed by radish and lettuce roots. However, the increase in lettuce roots is more gradual in relation to stems, with a notable rise observed at the highest concentrations, approaching the maximum value. These findings suggest that the tested substance may elicit varying responses across different plant tissues and species, indicating that the observed effects may be contingent upon the specific plant part and the concentration employed.

The allelopathic potential of plants is commonly assessed based on two main parameters: seed germination and target plant growth [41]. The inhibitory effect observed with *M. marina* EO was significantly more pronounced on the development of the aerial and root structures than on the process of germination. This finding aligns with the results of previous studies that have demonstrated germination to be a less sensitive indicator than seedling growth [42, 43].

Previous research has demonstrated that monoterpenes reduce radicle length and prevent germination in *Raphanus sativus* [44]. Other studies have demonstrated that volatile oils derived from plants with a high monoterpene content have the ability to inhibit the germination and subsequent growth of *R. sativus* seeds. The compounds α‐terpinene, χ‐pinene, α‐phellandrene, linalool, and limonene were identified as the specific inhibitors of radicle length and *R. sativus* seed germination [17]. Other studies have indicated that eugenol is an effective germination delaying agent for hybrid rice seeds [45].

The tested oil has a greater impact on the length of the lettuce and radish roots than on the aerial parts. This was previously corroborated by Kato‐Noguchi et al. [46], who indicated that root growth is a more sensitive indicator of phytotoxicity than hypocotyl growth. Furthermore, allelochemicals are highly active in the meristematic tissues that are involved in root growth [47]. The presence of terpenes and their non‐terpene derivatives may be responsible for this effect [17].

In general, lettuce and radish exhibited significant inhibition by the tested oil, with the extent of this inhibition varying according to the nature of the extract, its concentration, and the part of the plant [43].

### 2.4 Molecular Docking Results

#### 2.4.1 Molecular Docking Study Against Human Pancreatic α‐Amylase (PDB ID: 3BAJ)

In silico technologies are playing an increasingly pivotal role in drug discovery and development, primarily due to their expeditious throughput, cost‐effectiveness, and labor‐saving characteristics, particularly in comparison to their in vitro and in vivo counterparts for the identification of novel natural compounds as drug targets with predicted biological activity [48], computational methods by using molecular docking were performed. In light of the aforementioned studies, which employed docking simulations against “human pancreatic α‐amylase” [49, 50, 51], we conducted a docking study of the five predominant phytocompounds of *M. marina* EO. This was done to predict the mechanism of action associated with the results of the in vitro test.

**FIGURE 3:**
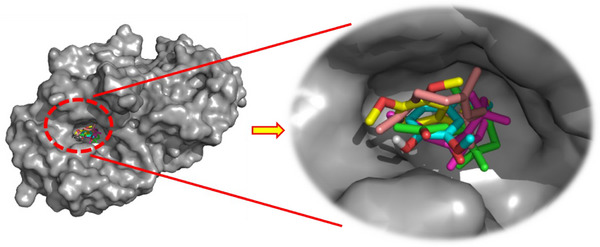
The 3D binding modes of the docked ligands: eugenol (cyan color), (*E*)‐β‐damascenone (purple color), methyl eugenol (yellow color), α‐humulene (pink color), and (*E*)‐β‐ionone (green color) in the binding cavity of human pancreatic α‐amylase (PDB ID: 3BAJ).

**FIGURE 4:**
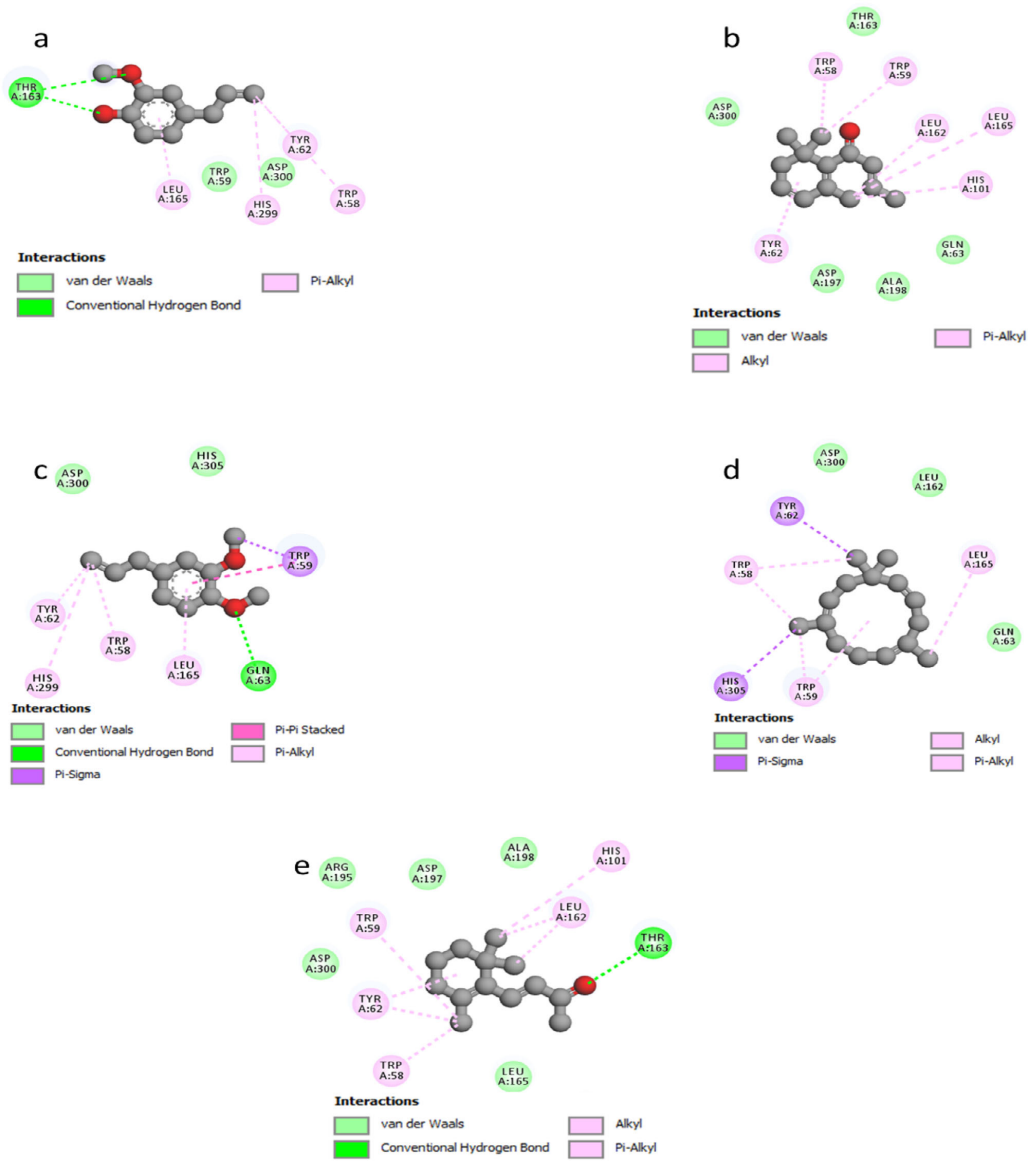
2D model of different interactions; (a) eugenol, (b) (*E*)‐β‐damascenone, (c) methyl eugenol, (d) α‐humulene, and (e) (*E*)‐β‐ionone within the active site of “human pancreatic α‐amylase” (PDB ID: 3BAJ).

As illustrated in Figure 3, the docked ligands exhibit a favorable fit within the receptor's binding cavity. As evidenced by the tabulated data, two of the docked ligands demonstrated a superior binding score compared to the standard “acarbose.” The most effective compound is α‐humulene, which had the best docking score (binding energy value = −7.2 kcal/mol). It is involved in some pi–sigma interactions with Tyr62 and His305, as well as alkyl/pi–alkyl contacts with Trp58, Trp59, and Leu165 (Figure 4d). As illustrated in Table 4, (*E*)‐β‐damascenone (−6.5 kcal/mol) was identified as the second most active ligand, exhibiting numerous alkyl/pi–alkyl interactions with the amino acid sequence: Trp58, Trp59, Tyr62, His101, Leu162, and Leu165 (Figure 4b). The absence of hydrogen bonds can be attributed to the stability of the ligand's docking complex, which is facilitated by hydrophobic interactions. This observation highlights the significance of these interactions in the inhibition of the targeted enzyme, human pancreatic α‐amylase. In contrast, (*E*)‐β‐ionone was identified as the third most bioactive phytocompound, exhibiting not only alkyl/pi–alkyl interactions but also a conventional H‐bond with Thr163 (Figure 4e). The fourth most effective anti‐α‐amylase molecule is methyl eugenol, which forms a conventional H‐bond with Gln63 in addition to other interactions detailed in Figure 4c. As illustrated in Figure 4a, despite the formation of two hydrogen bonds, eugenol was identified as the fifth most bioactive ligand based on the calculated docking scores. Based on the aforementioned results, it can be inferred that the five predominant constituents of *M. marina* EO possess a notable capacity to inhibit human pancreatic α‐amylase.

**TABLE 4:** Binding energy of the docked compounds in the binding cavity of human pancreatic α‐amylase (PDB ID: 3BAJ).

Compound	Binding energy (kcal/mol)
Eugenol	−5.2
(*E*)‐β‐Damascenone	−6.5
Methyl eugenol	−5.4
α‐Humulene	−7.2
(*E*)‐β‐Ionone	−5.9
Acarbose^R^	−6.3

Abbreviation: R, reference.

#### 2.4.2 Molecular Docking Study Against 4‐Hydroxyphenylpyruvate Dioxygenase (PDB ID: 6J63)

In our outcomes from the in vitro test, the tested *M. marina* EO was found to have good allelopathic potential against the selected species. 4‐Hydroxyphenylpyruvate dioxygenase (HPPD) was selected as a target receptor in this study. HPPD is an important target protein for herbicide development. This enzyme was selected as the target receptor because it is the molecular target for molecules with postemergence herbicidal activity. Therefore, in plants, the inhibition of this protein results in the depletion of carotenoids, and so the absence of chloroplast growth in emerging foliar tissues results in necrosis and death [52, 53].

**FIGURE 5:**
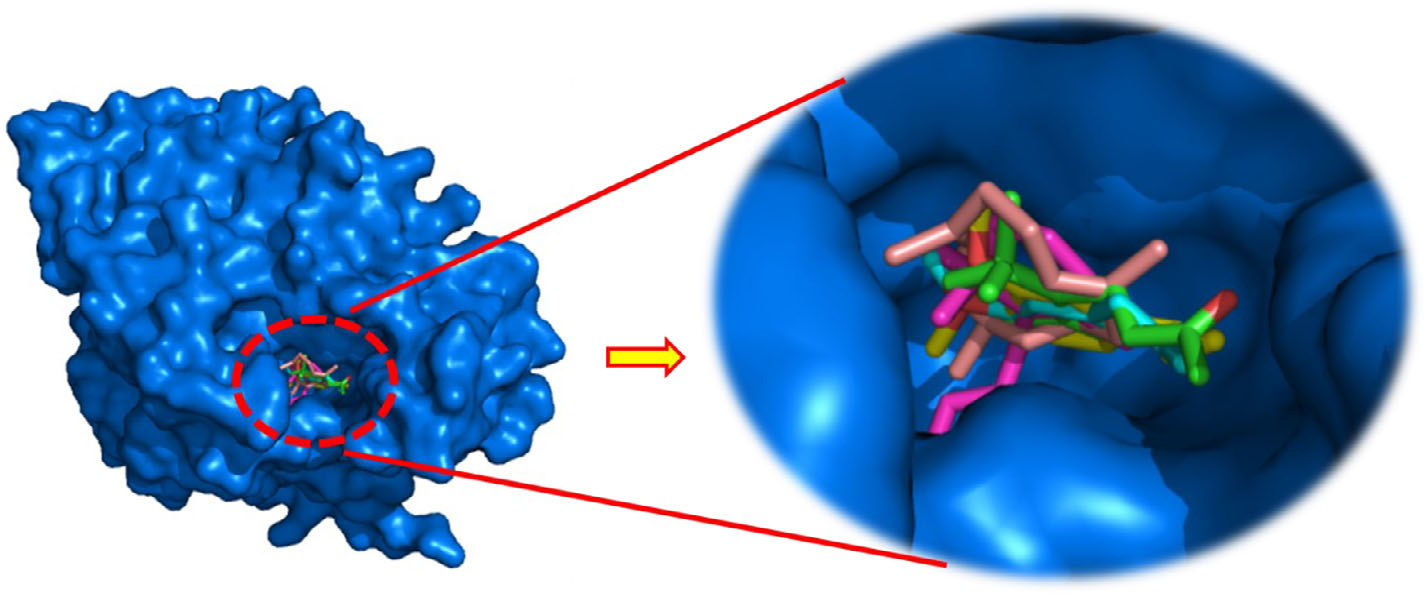
The 3D binding modes of the docked ligands: eugenol (cyan color), (*E*)‐β‐damascenone (purple color), methyl eugenol (yellow color), α‐humulene (pink color), and (*E*)‐β‐ionone (green color) in the binding cavity of HPPD.

**FIGURE 6:**
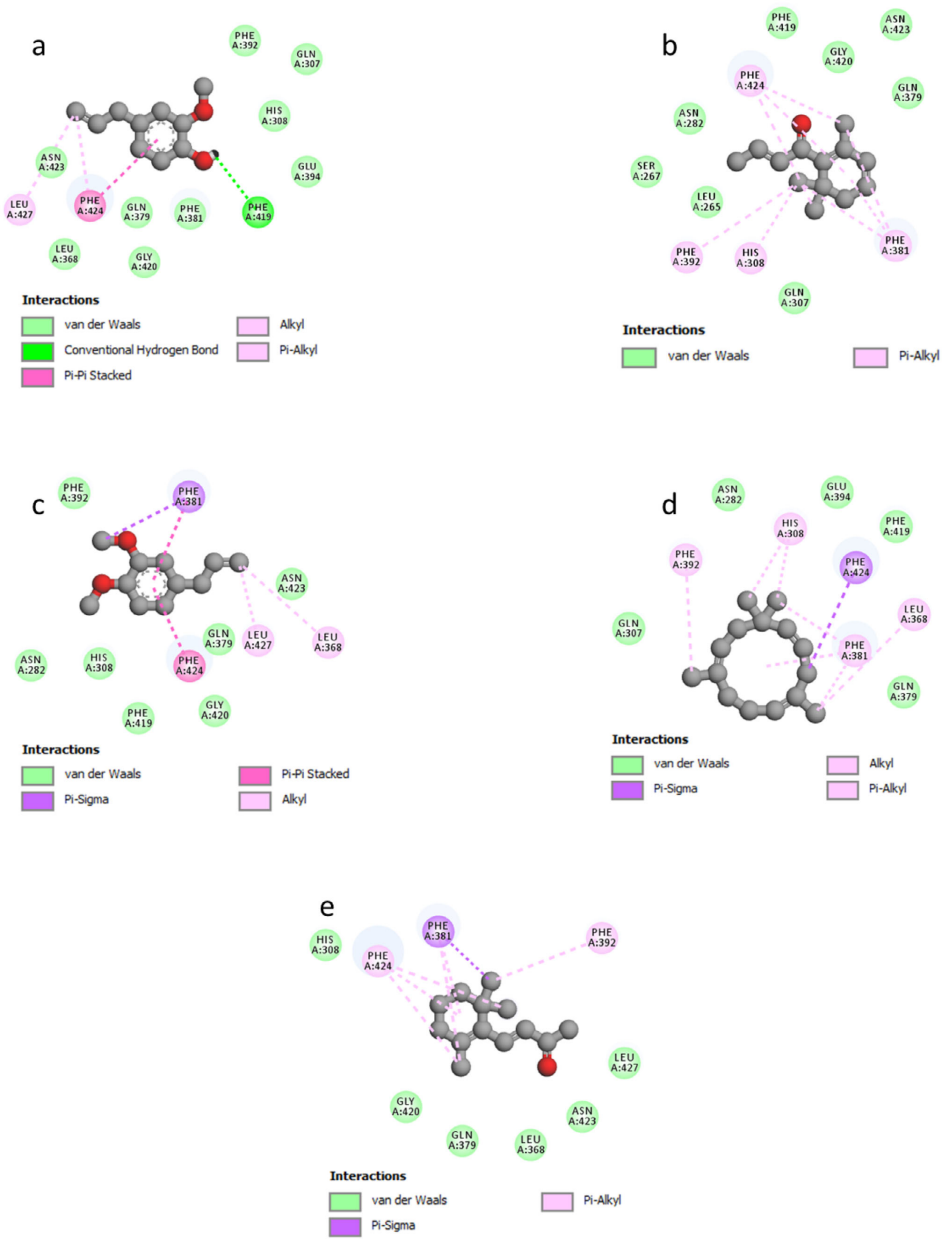
2D model of different interactions; (a) eugenol, (b) (*E*)‐β‐damascenone, (c) methyl eugenol, (d) α‐humulene, and (e) (*E*)‐β‐ionone within the active site of HPPD (PDB ID: 6J63).

As depicted in Figure 5, the docked ligands fit well in the receptor's binding cavity. Indeed, as Table 5 shows, among all docked ligands, (*E*)‐β‐ionone displayed the best binding affinity with HPPD (−6.7 kcal/mol). The inhibitory potential of this ligand is perceptible *via* the formation of a Pi‐Sigma interaction with Phe381 in addition to many P‐alkyl contacts with Phe381, Phe392, and Phe424. The second most effective ligand (*E*)‐β‐damascenone (−6.6 kcal/mol) is involved in several P‐alkyl interactions with residues: His308, Phe381, Phe392, and Phe424. α‐Humulene was found to be the third most bioactive phytocompound by displaying pi–sigma with Phe424‐alkyl/P‐alkyl with His308, Leu368, Phe381, and Phe392, while eugenol's potential is noticeable via the formation of a conventional H‐bond with Phe419 in addition to pi–pi stacked with Phe424 and some alkyl/P‐alkyl contacts with Phe424 and Leu427. Methyl eugenol was found to be the least active ligand by exhibiting some interactions detailed in Figure 6. All the above results show that the major constituents of *M. marina* EO have a significant tendency to inhibit HPPD.

**TABLE 5:** Binding energy of the docked compounds in the binding cavity of HPPD “PDB ID: 6J63.”

Compound	Binding energy (kcal/mol)
Eugenol	−5.6
(*E*)‐β‐Damascenone	−6.6
Methyl eugenol	−5.5
α‐Humulene	−6.4
(*E*)‐β‐Ionone	−6.7
Sulcotrione^R^	−7.5

Abbreviation: R, reference.

## 3 Materials and Methods

### 3.1 Plant Material

Aerial parts of *M. marina* were obtained from the island of Djerba, located in the south of Tunisia, during the reproductive and vegetative seasons in July and January, respectively. Prof. Fethia Skhiri, a botanist at the High Institute of Biotechnology in Monastir, Tunisia, performed taxonomic identification. A specimen was submitted to our laboratory at the Faculty of Pharmacy in Monastir, Tunisia (MM‐H).

### 3.2 EO Extraction

Fresh aerial parts (2 kg) during both seasons were hydrodistilled in a Clevenger‐style device for 4 h. The resulting oils (0.16% for the reproductive stage and 0.071% for the vegetative stage) were dried and stored in glass vials at 4°C–5°C until analysis.

### 3.3 GC–FID and GC–MS Analysis

Quantitative analysis was performed using an HP 5890 Series II gas chromatograph (Hewlett‐Packard, USA) equipped with a flame ionization detector (FID) and an HP‐5 capillary column (30 m × 0.25 mm i.d., 0.25‐µm film thickness). The oven temperature program started at 60°C (held for 10 min), followed by an increase of 5°C/min up to 220°C, and held at this final temperature for 10 min. The injector and detector temperatures were set at 240°C. Helium was used as the carrier gas at a constant flow rate of 2.0 mL/min. Injecarrier carried out in split mode (1:30), using 0.5 µL of a 10% hexane solution. Qualitative analysis was conducted using a gas chromatography–mass spectrometry (GC–MS) system consisting of an Agilent 7890A GC coupled to an Agilent 5975C mass selective detector. The system used an HP‐5 capillary column (30 m × 0.25 mm, 0.25‐µm film thickness). Injector and transfer line temperatures were maintained at 220°C and 240°C, respectively. The oven temperature was programmed from 60°C to 240°C at a ramp rate of 3°C/min. Helium served as the carrier gas, and 0.2 µL of a 10% *n*‐hexane solution was injected in split mode (1:30). Retention indices were calculated using a homologous series of *n*‐alkanes (C8–C40; Sigma‐Aldrich) based on the method proposed by Van den Dool and Kratz [54]. When available, authentic standards were used to confirm retention times. Compound identification was achieved through mass spectral matching using the NIST 2011 [55] library and a validated in‐house database [56].

### 3.4 α‐amylase Inhibition Activity

The α‐amylase inhibition assay was conducted in triplicate and in accordance with the methodology previously described by Ali et al. [57], with certain modifications. The concentrations of the EOs used ranged from 0.009 to 1.25 mg/mL.

Ten microliters of the EO were combined with 50 µL of the α‐amylase solution (0.5 mg/mL) with 0.02 M sodium phosphate buffer (pH 6.9 with 0.006 M NaCl) in a 96‐well plate and incubated for 10 min. After the preincubation phase, a solution of starch (1%) in 0.02 M sodium phosphate buffer (pH 6.9 with 0.006 M sodium chloride) was then added (100 µL), and the mixture was incubated for 3 min at 25°C. The reaction was finished with 125 µL of a 3,5‐dinitrosalicylic acid reagent, placed in a boiling water bath for 5 min, cooled to room temperature, diluted with 200 µL of deionized water, and the absorbance was then determined at 540 nm.

Furthermore, the absorbances of the control samples, which were prepared with a buffer solution in place of the extract and amylase, were determined, and acarbose was used as the positive control with the same concentration as the EO. The calculation of amylase inhibition was performed using the following equation:

$$\%\;\alpha -\mathrm{Amylase}\;\mathrm{inhibition}=\left(\frac{OD_{\mathrm{control}}-OD_{\mathrm{sample}}}{OD_{\mathrm{control}}}\;\right)\times 100$$

where OD_control_ is the optical density of the control (untreated sample) and OD_sample_ is the optical density of the treated sample.

### 3.5 Determination of the Allelopathic Activity

#### 3.5.1 Seed Germination

The EOs of *M. marina* was evaporated in chloroform, and the volume was adjusted to get the desired concentrations. After inserting filter paper discs of Whatman no. 1 (*d* = 5.7 cm) in each Petri dish (*d* = 6 cm, *h* = 1 cm), 0.5 mg/mL of the obtained oils was applied. After the solvent had been dissolved at room temperature (24 h), 10 seeds were put in each Petri dish [17, 43]. Distilled water served as negative control. The criterion for germination reading was the rootlet protrusion. The experiment was performed in triplicates. The percentages of germination inhibition were calculated by comparison with the untreated control, using the following calculation:

$$\%\;\mathrm{Germination}\;\mathrm{inhibition}=\left(\frac{C-X}{C}\right)\times 100$$

where *C* is the number of seeds germinated in the control and *X* is the number of seeds germinated in the test sample.

#### 3.5.2 Roots and Stem Growth

To evaluate the impact of EOs on the growth of stem sand roots, the inhibition percentages were determined after 7 days. The findings were then compared to those of the untreated control using the following formula:

$$\%\;\mathrm{Growth}\;\mathrm{inhibition}=\left(\frac{C-X}{C}\right)\times 100$$

where *C* is the average size of the root/stem in the control group, *X* is the average size of the root/stem in the test sample.

The results are presented as follows. The standard deviation value for the untreated control was also considered, and the experiment was established in triplicate.

### 3.6 Molecular Docking Procedure

Before docking, the crystal structures of the enzymes, “human pancreatic α‐amylase in complex with nitrate and acarbose ‘PDB ID: 3BAJ’ [55] and ‘*Arabidopsis thaliana*’ HPPD complexed with NTBC ‘PDB ID: 6J63’” [56], were downloaded from the RSCB protein data bank (https://www.rcsb.org/). The in silico studies were conducted using the AutoDock 4.2 program package [58]. The tested compounds were constructed and subsequently optimized using the ACD three‐dimensional viewer software (ACD/Labs. ACD/3D Viewer, version 2017.2.1) [59]. In preparation for the docking process, all missing hydrogens and Gasteiger charges were added to the receptor input file. The ligands and protein files were prepared using AutoDock Tools in PDBQT format. Furthermore, the grid maps were generated using AutoGrid, which proved an efficient method for reducing the time required for the docking steps. Three‐dimensional affinity grids of 0.375 Å spacing were centered on the receptor to determine the active site. The graphical material was prepared using Discovery Studio 2017 R2 (BIOVIA, Dassault Systems, Discovery Studio Visualizer, (v17.2.0.16349) (Dassault Systems, 2017)) [60] and PyMOL 0.99rc6 [61] and the PyMOL Molecular Graphics System (Version 0.99rc6. Schrödinger, LLC, De Lano Scientific, San Carlos). The docking outcomes are based on the binding energy (kcal/mol) and intermolecular interactions.

### 3.7 Statistical Analysis

All data are presented as mean ± standard deviation. IBM SPSS Statistics 20 was used, and the data were evaluated using one‐way ANOVA and the Tukey test. A *p* value of less than 0.05 was considered statistically significant.

## 4 Conclusions

Our study has investigated the difference between the chemical composition of EOs when comparing the two stages of plant development that had a direct impact on their biological activities. This compositional variation was associated with enhanced amylase inhibitory action during the reproductive stage (87.33% of inhibition at 1.25 mg/mL), which was further supported by in silico molecular docking analysis showing stronger binding affinities of key compounds to the enzyme's active, which makes it an effective glucose‐lowering plant‐derived agent., In addition, *M. marina* EO demonstrated interesting inhibitory effects on lettuce and radish seeds germination as well as stems and roots. Further studies are needed to investigate the allelopathic mechanism of action of the volatile and hydro‐soluble compounds, and increase the likelihood of using them as bioherbicides in the future. More research will be required.

## Conflicts of Interest

The authors declare no conflicts of interest.

## Data Availability

Data is contained within the article.
